# Stachyose supplementation attenuates ischemic stroke injury via gut-brain axis mediated adenine metabolism and neuroactive ligand-receptor signaling

**DOI:** 10.1016/j.crfs.2026.101556

**Published:** 2026-09-10

**Authors:** Zichen Zhang, Haoyan Zhang, Xueying Liu, Shengxiang Wang, Yichen Liu, Peng Liu, Shangyong Li, Jieting Zhang, Ningning He, Shihai Liu

**Affiliations:** a Medical Animal Lab, Medical Research Center, The Affiliated Hospital of Qingdao University, Qingdao, Shandong, 266500, China; b Department of Pharmacy, Qingdao Central Hospital, University of Health and Rehabilitation Sciences, Qingdao, 266042, China; c School of Basic Medicine, Qingdao University, Qingdao, 266000, China

**Keywords:** Stroke, Stachyose, Gut-brain axis, Gut microbiota, Multi-omics, Neuroactive ligand-receptor interaction, Neuroprotection

## Abstract

Stroke is a neurological disease with high incidence and severe disability. Stroke recovery is closely associated with the homeostasis of the gut-brain axis and the regulation of neuroinflammation and neuronal function. As a prebiotic with potent gut microbiota-regulating and anti-inflammatory activities, stachyose (STA) has been increasingly recognized for its beneficial effects on host health. To explore the neuroprotective effects of STA on stroke and its underlying mechanisms, we established a photothrombotic stroke mouse model and performed integrated multi-omics analyses, including 16S rRNA sequencing and metabolomics of feces, as well as metabolomics and transcriptomics of brain tissue. The 16S rRNA analysis showed that STA treatment significantly reshaped the gut microbial community structure in mice. Metabolomics of brain and feces further identified brain-gut shared metabolites, which may act as signaling molecules in the gut-brain axis, and these key metabolites were significantly correlated with specific microbial taxa and critical genes. Transcriptomic analysis of brain tissue revealed that STA treatment markedly regulated the neuroactive ligand-receptor interaction pathway, which is crucial for the synthesis, release and binding of neurotransmitters, as well as for neuronal survival, differentiation and maintenance of neural function. Collectively, STA may exert neuroprotective effects in stroke by regulating gut microbiota-associated metabolites and subsequently modulating the neuroactive ligand-receptor interaction signaling pathway, thereby mediating crosstalk along the gut-brain axis.

## Highlights

• Stachyose (STA) shows potent neuroprotective activity in mice with photothrombotic ischemic stroke.
• STA reshapes disturbed gut microbiota and modulates shared gut-brain metabolites of stroke mice.
• Multi-omics uncovers key microbes, metabolites and hub genes mediating gut-brain axis crosstalk.
• STA alleviates stroke damage through microbiota-metabolite axis and neuroactive ligand-receptor pathway.

## Graphical abstract

**Figure:**
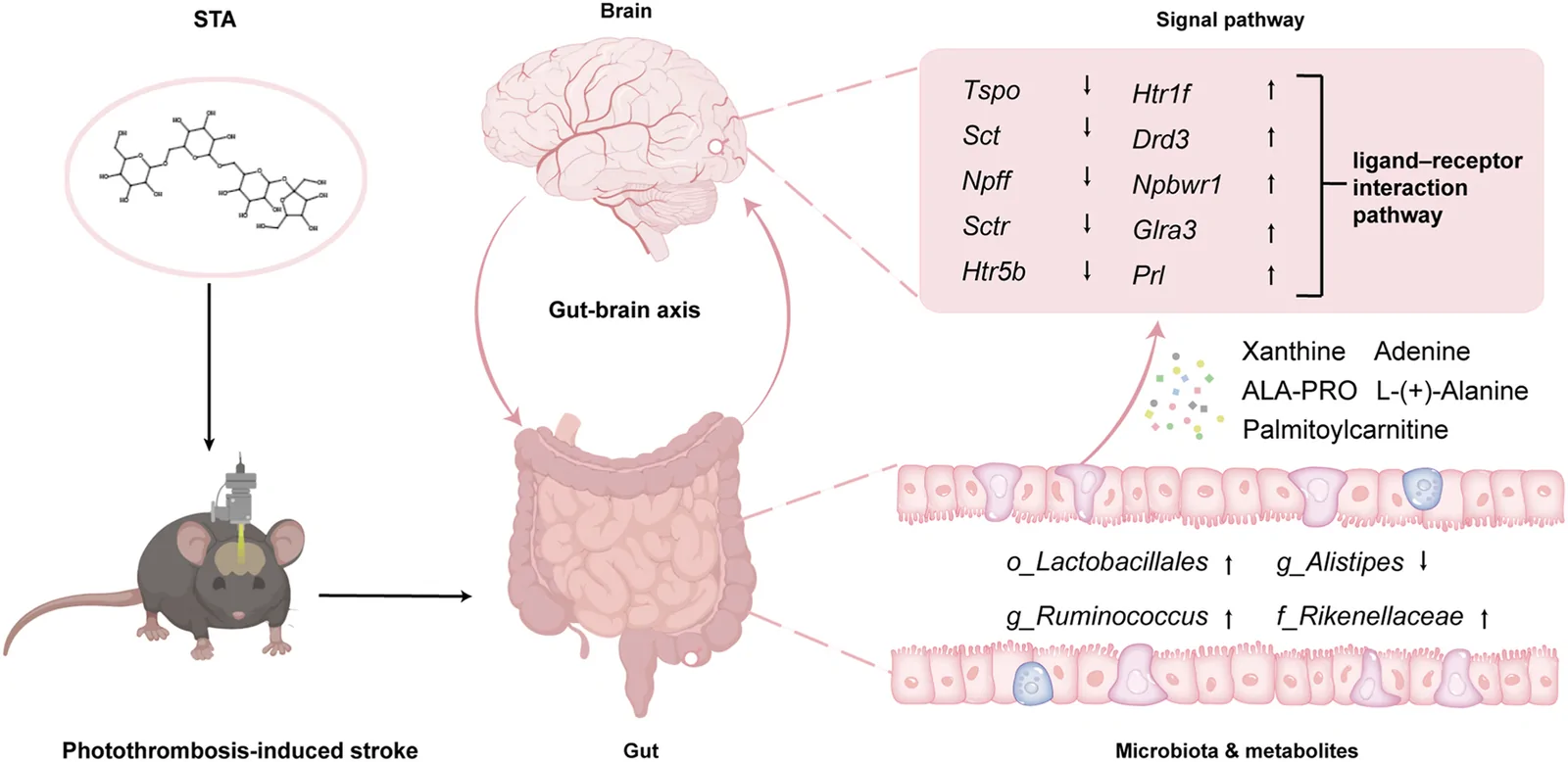

## 1 Introduction

Acute ischemic stroke is a medical emergency caused by the reduction of blood flow to the brain, which might lead to nerve function problems and Brain-related symptoms (Walter, 2022). Stroke is the second leading cause of death worldwide. It is projected that a growing proportion of global stroke death will be caused by intracerebral hemorrhage (accounting for 44.3% in 2020 and 53.4% in 2050) (Feigin and Owolabi, 2023). Current modern medical guidelines for stroke in various countries, including medical thrombolysis, surgical thrombectomy and anticoagulation therapy, all impose restrictions regarding the therapeutic time window (Saini et al., 2021). Due to the high mortality rate of the ischemic stroke, its increasing prevalence year by year, and the limitation of the current treatment methods, there is an urgent need to find more effective therapeutic approaches.

In recent years, accumulating evidence has demonstrated that the gut microbiota can influence intestinal metabolites through its metabolic activities and other biological functions, thereby modulating brain physiological processes via the gut-brain axis. Among these mechanisms, microbiota-derived metabolites are considered key signaling molecules mediating communication between the gut and the brain (Deng et al., 2024; Kim, 2024). Moreover, accumulating studies indicate that the gut microbiota and their metabolites contribute to neuroinflammation and cognitive dysfunction in neurological disorders, including Alzheimer's disease (Dilmore et al., 2023; Liu et al., 2020), Parkinson's disease(Mahbub et al., 2024), and depression (Zhu et al., 2022). Collectively, these gut-derived metabolites can influence the metabolic state of brain tissue and regulate neuroinflammation, blood-brain barrier integrity, and neural signaling pathways, thereby affecting gene expression and neurological function. Therefore, the gut microbiota-metabolite-brain signaling axis is increasingly recognized as an important mechanism linking intestinal microbial ecology with central nervous system regulation.

Prebiotics are non-digestible food components that benefit the host by selectively stimulating the growth and/or activity of specific gut microbiota (Schoemaker et al., 2022; Xi et al., 2020). Stachyose (STA) is a naturally occurring tetrasaccharide belonging to the raffinose family oligosaccharides (RFOs). RFOs are widely distributed in plants, particularly in legume seeds such as soybean. Due to the limited α-galactosidase activity in the human gastrointestinal tract, stachyose escapes digestion and absorption in the small intestine and reaches the colon, where it is fermented and metabolized by gut microbiota (Elango et al., 2022; Li et al., 2017). As a prebiotic, STA can modulate gut microbial composition and metabolic activity, thereby altering microbial-derived metabolites that may influence host physiological functions (He et al., 2024, 2026; Kim et al., 2025). Recent evidence further shows that STA ameliorates stress-induced hippocampal long-term potentiation (LTP) impairment and restores cognitive function through modulation of the gut microbiota (Y. Huang et al., 2022). Together, these findings suggest that STA may influence the central nervous system via the gut-brain axis by regulating gut microbial composition and metabolism. However, its role in stroke remains largely unexplored and requires further systematic investigation.

In the present study, we employed a mouse model of photothrombotic stroke to investigate the potential mechanisms underlying the neuroprotective effects of STA supplementation against ischemic brain injury. To systematically dissect the underlying regulatory network, we conducted integrative multi-omics analyses, including 16S rRNA gene sequencing and metabolomics of fecal samples, as well as metabolomics and transcriptomics of brain tissue. Our findings may offer a multi-omics basis for understanding microbiota-dependent metabolic signaling in ischemic brain injury.

## 2 Material and methods

### 2.1 Materials

Rose bengal (CAS 632-69-9, molecular weight: 1017.64 g/mol) was purchased from Sigma. The ELISA kits for IL-1β, IL-6, TNF-α, and IL-10 were purchased from Thermo Fisher Scientific (Waltham, MA). TTC (2,3,5-triphenyltetrazolium chloride, CAS 298-96-4) staining solution was obtained from Servicebio (Wuhan, China, G1017-100 ML). The solution was stored at 4 °C in the dark and transported on wet ice according to the manufacturer's instructions. STA hydrate (Cat# A9110, 25 g) was purchased from Solarbio. Isoflurane (veterinary prescription drug for pets) was purchased from RWD. The product was manufactured under Chinese Veterinary Drug GMP (Certificate No. 15125) with manufacturing license No. 15198.

### 2.2 Animal experiment

The animal experiment protocol adhered to the NIH guidelines and received approval from the Ethics Committee of Medical College of Qingdao University (Approval No.QDU-AEC-2024780). Male C57BL/6J mice (6 weeks old, 18-20 g) were maintained under standard laboratory conditions: a 12-h light/dark cycle and free access to food and water in an air-conditioned room.

The sample size was determined using G*Power 3.1 (one-way ANOVA, α = 0.05, power = 0.80, effect size f = 0.40), with reference to previous photothrombotic stroke studies. Animals were excluded only when the photothrombotic stroke model was unsuccessfully established or when samples failed quality control criteria for subsequent analyses.Following a 7-day acclimatization period, mice were randomly allocated into 3 groups (n = 8): normal drinking water control group (NC), photothrombosis-induced stroke group (PT), and STA treatment group (STA). Mice in the NC and PT groups were given purified water for 14 days, while mice in the STA group were given STA supplementation (400 mg/kg/day)(Kim et al., 2025). STA was administered by oral gavage once daily at a volume of 0.1 mL per mouse, with the dose adjusted according to individual body weight. The dose of STA was referenced from doses used in other experimental models and further determined based on our preliminary pre-experiments. In the third week, the NC group underwent sham operation, whereas the PT and STA groups received photothrombosis therapy. Subsequently, the STA group received STA treatment for the following 7 days. Throughout the intervention, body weight was recorded daily. On the final experimental day, the mice first underwent the grid walking test and the cylinder test. Subsequently, all mice were anesthetized using inhalation anesthesia. Retro-orbital blood samples were collected first, followed by euthanasia. Brain tissue and fecal samples were then harvested. Blood samples were centrifuged to obtain serum.

### 2.3 Photothrombotic (PT) stroke model

Mice were intraperitoneally injected with rose bengal (10 mg/mL, 10 mL/kg body weight)(Lv et al., 2018). Following injection, the mice were anesthetized and secured in a stereotaxic apparatus using ear bars and a nose bar, ensuring that the line connecting both ears remained horizontal. Subsequently, incise the scalp along the midline and use a dry cotton swab to peel back the periosteum, exposing the skull. Using the anterior fontanelle as the origin point, designate the location 1.5 mm to the right of this origin as the laser irradiation site. Activate the green laser source. After 20 min of laser irradiation, disinfect and suture the skin incision. Place the mouse on a constant-temperature heating pad to await recovery.

### 2.4 TTC staining

Brain tissue was placed in a pre-chilled brain mold and sectioned into 2-mm-thick coronal slices. The slices were placed in a 0.5% TTC solution preheated to 37 °C and incubated in a 37 °C incubator for 10 min; the brain sections were then flipped to the other side and incubated for an additional 5 min. Upon completion of staining, the infarcted areas appeared pale, while normal brain tissue appeared bright red. The stained brain sections were fixed in 4% paraformaldehyde for 6 h, then arranged in order, and both sides of the sections were scanned.

### 2.5 Cylinder test and grid walking test

In the cylinder test, each mouse was placed in a glass beaker and recorded from above for 5 min. As the mouse naturally reared to explore the container, the forelimb used to make contact was recorded as either “left” or “right”, and if both forelimbs were used simultaneously or with lateral movements to move along the wall, a recording of “both” was used. In the instances where one forelimb made contact first, and then the other forelimb made contact without the removal of the first forelimb, an “independent” and “both”recording were used. The results are expressed as a percentage of (ipsilateral/uninjured) forelimb used relative to the total number of contacts.

The grid walking test is used to measure gross motor impairments. Mice were placed on a raised wire grid (39 cm × 25.5 cm) with 1 cm between wires and recorded for 5 min. The number of foot faults were scored for each forelimb and hindlimb. A foot fault was determined as any step where the foot went through a grid hole or when the animal was resting with the wrist at the same level as the grid. The results are expressed as a percentage of (contralateral/injured) foot faults.

### 2.6 Enzyme-linked immunosorbent assay (ELISA)

The whole blood specimens were centrifuged at 3000 rpm for 30 min at 4 °C and serum was extracted from the blood. According to the manufacturer's instructions, the ELISA kit was used to detect the serum levels of TNF-α, IL-1β and IL-6.

### 2.7 16S rRNA sequencing

Genomic DNA was extracted from the collected fecal samples for PCR amplification and 16S rRNA sequencing following a previously described protocol (Wang et al., 2022). Briefly, microbial community diversity and composition were evaluated based on Bray–Curtis dissimilarities. Principal coordinate analysis (PCoA) was performed to visualize differences in microbial community structure among experimental groups. Overall differences in community composition among groups were assessed using permutational multivariate analysis of variance (PERMANOVA) based on Bray-Curtis distances (Table S1), and pairwise PERMANOVA was further performed to identify differences between individual experimental groups (Table S2). Permutational analysis of multivariate dispersions (PERMDISP) was conducted to evaluate whether differences in within-group community dispersion contributed to the observed PERMANOVA results (Table S3). Differentially abundant microbial taxa were identified using the analytical workflow described previously (H. Wang et al., 2022).

### 2.8 Transcriptome analysis and metabolomics analysis

Total RNA extracted from the collected samples was subjected to library construction and transcriptome sequencing according to a previously described protocol (Mu et al., 2025). Metabolites were extracted from the collected samples and subjected to untargeted metabolomic profiling using liquid chromatography-mass spectrometry, with all procedures performed according to a previously reported protocol (Liu et al., 2025).

### 2.9 Quantitative real-time PCR (RT-qPCR) analysis

The total RNA of liver tissues was isolated using TRIzol reagent following the manufacturer's instructions. The extracted RNA was then transcribed into cDNA (SweScript All-in-One RT SuperMix, Servicebio, Wuhan, China). Gene expression was measured using the 2×Universal Blue SYBR Green qPCR Master Mix (Servicebio, Wuhan, China), with Actb serving as the internal reference gene. The following primers were used for indicated genes as shown in Table S4.

### 2.10 Immunofluorescence staining

Formalin-fixed paraffin-embedded (FFPE) sections were incubated at 4 °C overnight with an anti-ADCY5 antibody (1:1000; Servicebio). After washing, sections were incubated at room temperature for 1 h with an iF555-Tyramide-labeled anti-rabbit IgG secondary antibody (1:500). Nuclei were counterstained with DAPI (G1012, Servicebio, 1:500). Images were acquired on a P250 FLASH scanner and spectrally unmixed using CaseViewer software (3DHISTECH).

### 2.11 Statistical analysis

Statistical analyses were performed utilizing GraphPad Prism (Version 9.5.1) and R software. The data are presented as mean ± stan dard deviation (SD) or mean ± standard error of the mean (SEM). Group differences were assessed using the unpaired Student's t-test, one-way ANOVA, two-way ANOVA, Kruskal-Wallis test, or Mann-Whitney *U* test (for non- normally distributed data). Associations between variables were evaluated via Spearman correlation analysis. Statistical significance was defined as *P* < 0.05.

## 3 Result

### 3.1 STA supplementation improves neurological outcomes in a photothrombotic stroke model

**Fig. 1:**
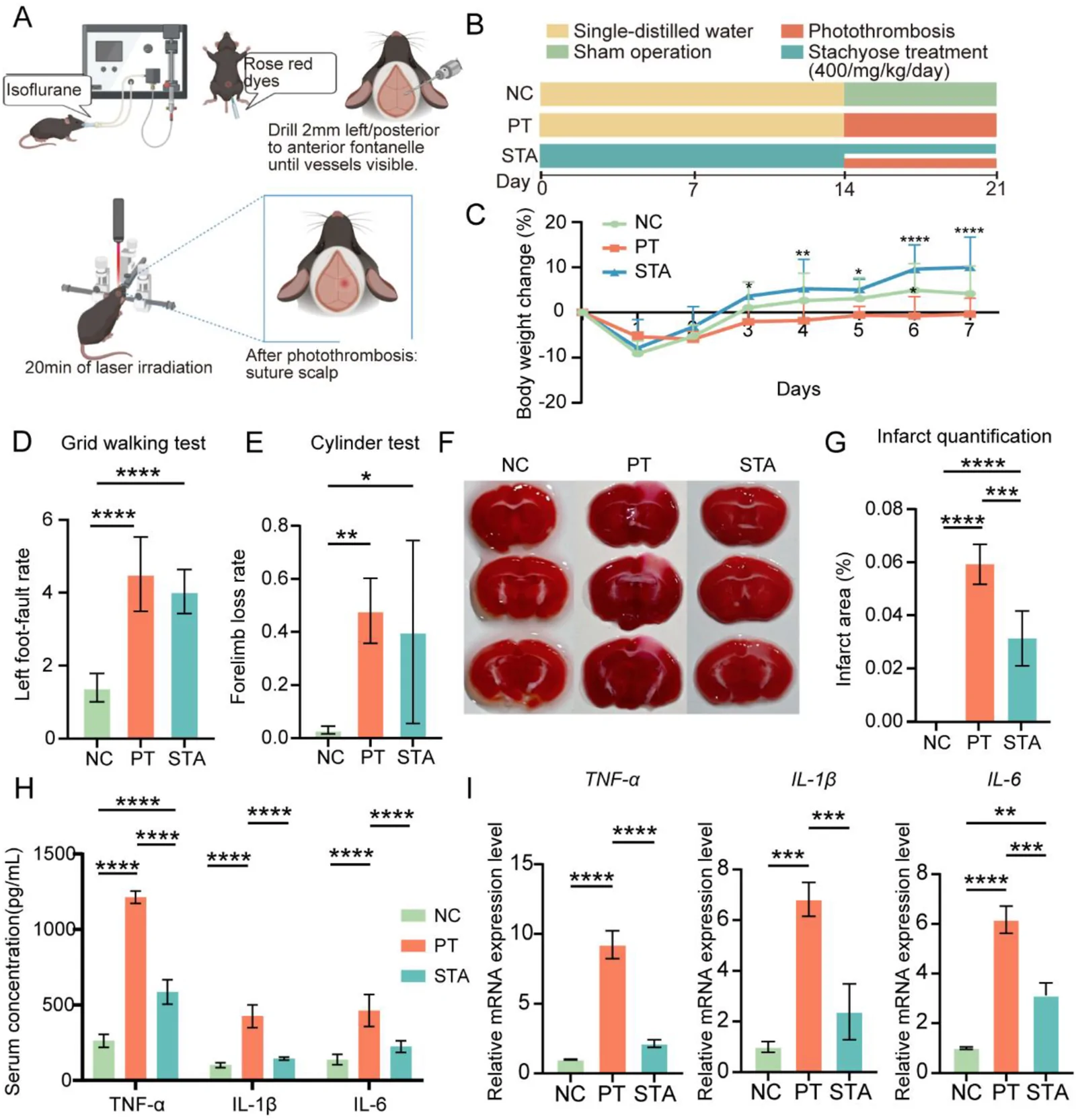
STA administration significantly attenuates **clinical symptoms in photothrombosis-induced stroke mice.** (n = 8) (A) Schematic illustration of the photothrombosis-induced stroke model. (B) Experimental workflow of the study. (C) Percentage change in body weight relative to the initial body weight on day 0. (D) Left hindlimb foot-fault rate in the grid walking test on the final experimental day. (E) Forelimb loss rate in the cylinder test on the final experimental day. (F) Representative brain sections showing infarct lesions detected by TTC staining. (G) Quantification of cerebral infarct area based on TTC-stained brain sections. (H) Serum concentrations of TNF-α, IL-6, and IL-1β. (I) The expression level of *TNF-α*, *IL-6*, and *IL-1β* in brain tissues. *P < 0.05, **P < 0.01, ***P < 0.001.****P < 0.0001. Data are presented as mean ± SD.

Consistent with the motor dysfunction and inflammatory responses observed in patients with ischemic stroke (Aldridge et al., 2023; Shichita et al., 2023), we established a mouse model of focal cerebral ischemia via photothrombosis (Fig. 1A). To evaluate whether STA supplementation exerts protective effects against post-stroke injury, mice received photothrombotic (PT) induction, and the overall experimental design is summarized in Fig. 1B. Compared with the NC group, mice in PT group showed a significant decrease in body weight. STA supplementation significantly attenuated this weight loss (Fig. 1C). Behavioral tests further supported the protective effect of STA. In the grid-walking test, PT mice exhibited a significantly higher contralateral hindlimb foot-fault rate, indicating impaired motor coordination. STA supplementationmarkedly reduced the foot-fault rate (Fig. 1D). In the cylinder test, the ratio of affected forelimb use to unaffected forelimb use was recorded to assess motor asymmetry. mice receiving STA supplementation showed improved forelimb use compared with the PT group (Fig. 1E). To evaluate infarct formation, brain sections were subjected to TTC staining. As shown in Fig. 1F, the PT group displayed a clear infarct area, whereas STA treatment significantly reduced infarct volume. Quantitative analysis further confirmed that STA supplementation significantly reduced the infarct area compared with the PT group (Fig. 1G).

To further assess inflammatory responses, serum levels of TNF-α, IL-6, and IL-1β were measured by ELISA (Fig. 1H). Compared with the NC group, these pro-inflammatory cytokines were significantly elevated in PT mice. STA supplementation significantly decreased their levels, suggesting that it attenuates stroke-induced systemic inflammation. Consistently, RT-qPCR analysis of brain tissue demonstrated that STA treatment reduced the expression of pro-inflammatory cytokines in the brain (Fig. 1I). Taken together, these results indicate that STA supplementation improves motor function, reduces infarct size, and suppresses both systemic and cerebral neuroinflammation in photothrombotic stroke mice, demonstrating its neuroprotective effect.

### 3.2 STA supplementation modulates the gut microbiota in a photothrombotic stroke model

**Fig. 2:**
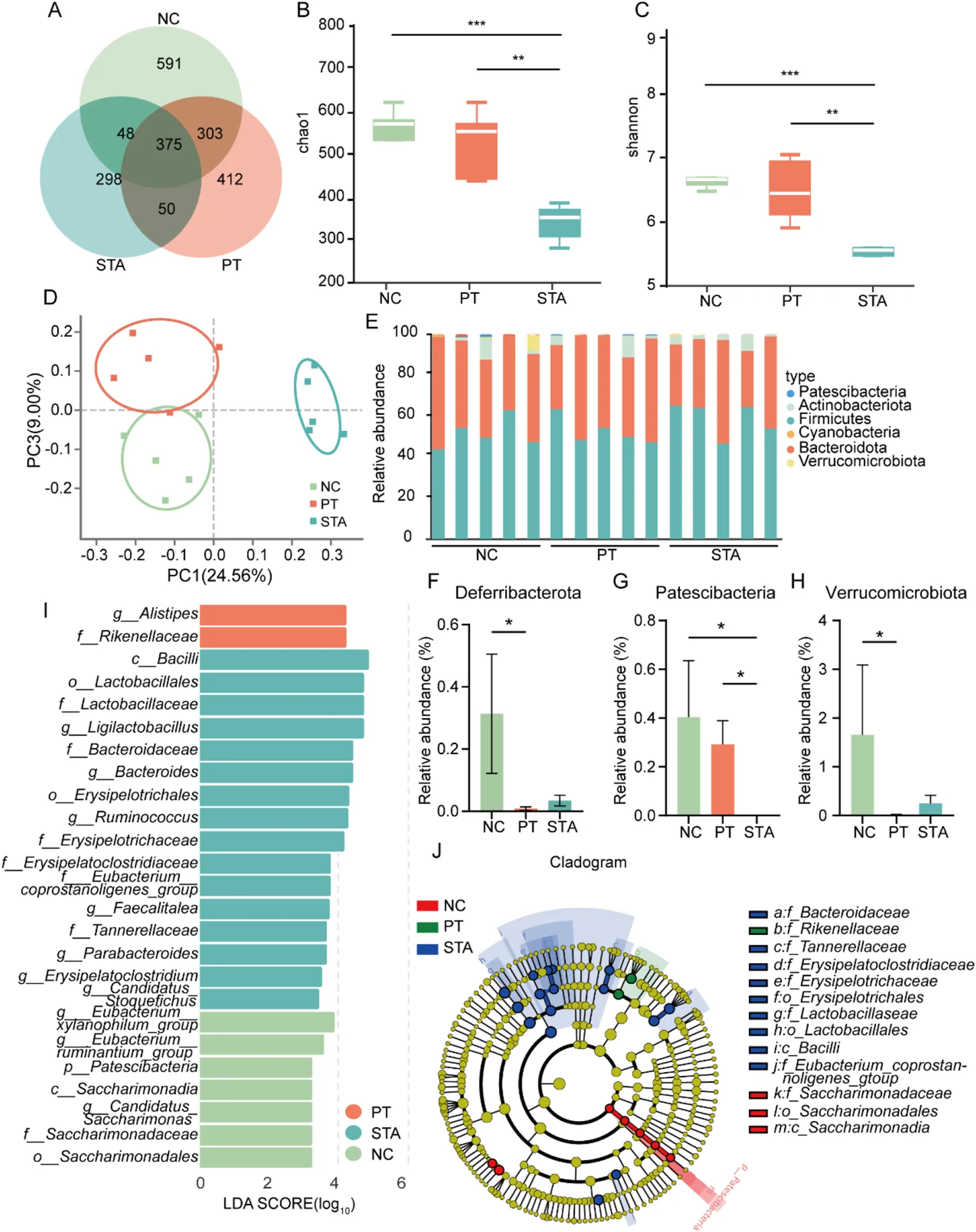
STA supplementation reshapes the gut microbiota dysbiosis in photothrombosis-induced stroke mice. The 16S rRNA sequencing analysis of stoke mice treated with STA. (n = 5). (A) The OUT Venn diagram based on 16S rRNA sequencing. (B) chao1 index indicating changes in α-diversity. (C) shannon index indicating changes in α-diversity. (D) Weighted UniFrac PCoA analysis. (E) The phylum-level organization of the gut microbiota. (F-G) The relative abundance of Patescibacteria (F) Verrucomicrobiota (G) and Defferribacterota (H). (I J) Distribution histogram (I) and cladogram (J) based on LEfSe analysis. Family (f), genera (g), class (c), phylum (p), and order (o) with *P* < 0.05 and LDA score (log_10_) > 2 were considered significant. Compared to the indicated group, *P < 0.05, **P < 0.01, ***P < 0.001. Data are presented as mean ± SEM.

To investigate the effect of STA supplementation on gut microbial composition, fecal samples collected from mice were subjected to 16S rRNA gene sequencing. The Venn diagram revealed differences in shared and unique OTUs among groups, suggesting alterations in gut microbial composition following STA supplementation. (Fig. 2A). Subsequently, the Chao1 and Shannon indices were used to evaluate the effect of STA on the α-diversity of the gut microbiota (Fig. 2B and C). The results showed that α-diversity was significantly reduced in the STA group. This finding suggests that STA supplementation altered the richness and evenness of gut microbial communities, potentially reflecting selective enrichment or depletion of specific bacterial taxa.The PCoA plot revealed a clear separation of the microbial communities in the STA group from those in the NC and PT groups (Fig. 2D), indicating that STA supplementation was associated with alterations in gut microbial community structure.

To further evaluate the specific effects of STA on gut microbial composition, taxonomic changes at the phylum level were analyzed (Fig. 2E–H). The results indicated that photothrombotic stroke markedly reduced the relative abundance of Patescibacteria, Verrucomicrobiota, and Deferribacterota, whereas STA treatment partially reversed these stroke-associated alterations. Linear discriminant analysis effect size (LEfSe) was subsequently applied to identify microbial taxa with significant differences at the phylum (p), class (c), order (o), family (f), and genus (g) levels. A total of 25 bacterial taxa were identified as significantly different among groups, suggesting that these microbes may serve as potential microbial biomarkers associated with disease progression (Fig. 2I and J). LEfSe analysis showed significant differences in gut microbiota composition among the groups. The g*_Alistipes* and its related taxon *f_Rikenellaceae* were significantly enriched in the PT group, whereas the STA group exhibited enrichment of several Firmicutes-related taxa, including *g_Ligilactobacillus, f_Lactobacillaceae, o_Lactobacillales, g_Ruminococcus,* and the *f_Eubacterium_coprostanoligenes_group*. In addition, *g_Bacteroides* and *g_Parabacteroides* were also enriched in the STA group, suggesting that STA intervention reshaped the gut microbial community composition. In contrast, *g_Alistipes* and its related taxa enriched in the PT group exhibited a decreasing trend in the STA group. These results indicate that STA supplementation altered gut microbial composition in stroke model mice, characterized by reduced abundance of *g_Alistipes* and increased abundance of several taxa, including *o_Lactobacillales*, *g_Ruminococcus*, and *g_Bacteroides*. Together, these findings suggest that STA modulates gut microbial community composition following ischemic injury.

### 3.3 STA supplementation alters gut metabolic profiles in photothrombosis-induced stroke mice and may influence brain metabolism

**Fig. 3:**
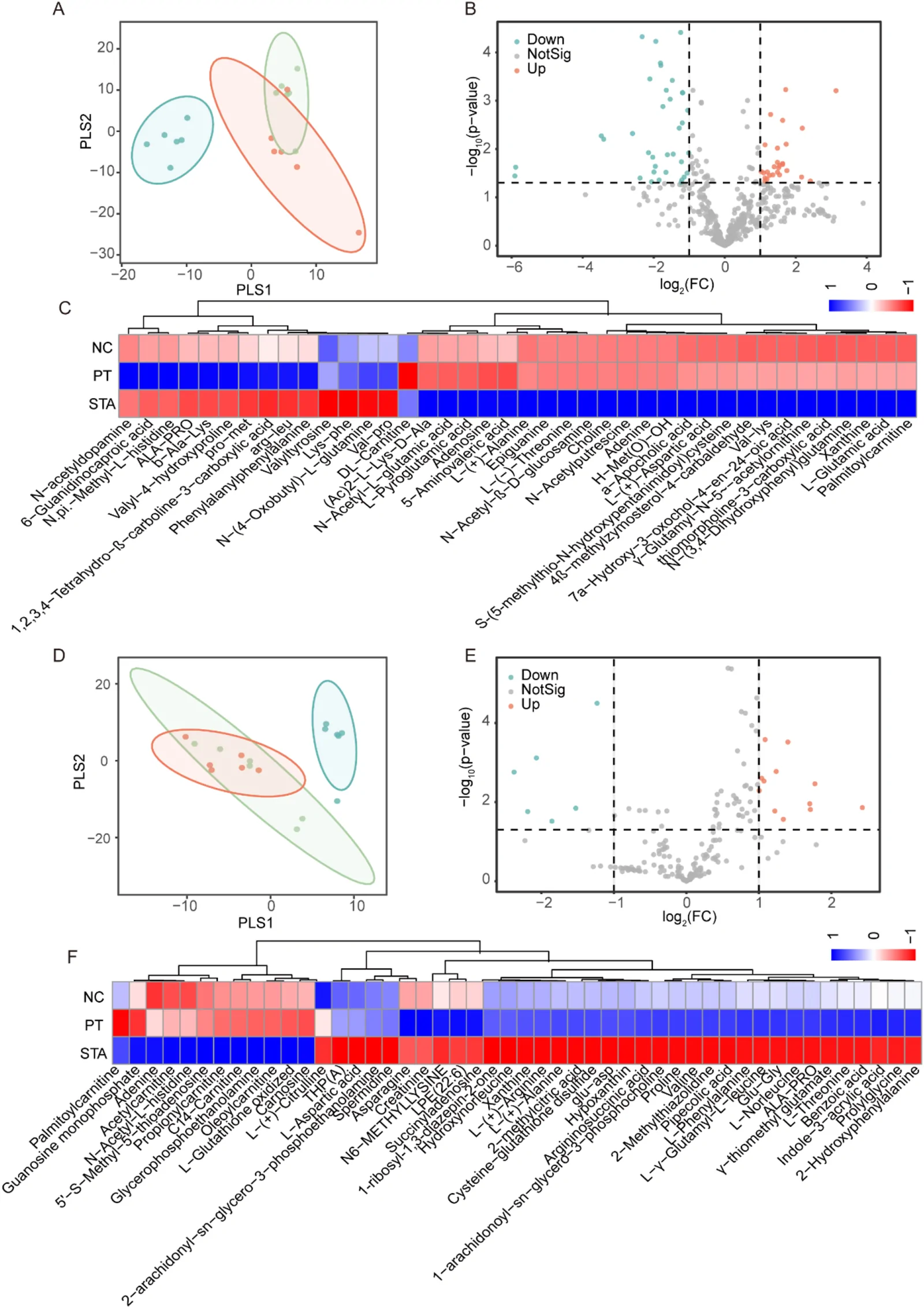
**STA supplementation alters intestinal and brain metabolites.** (n = 6) (A) PLS-DA analysis in positive of fecal metabolites. (B) Volcano plot showing differential metabolites between the STA and PT groups. (C) Heatmap showing correlations between differential metabolites and phenotypic indicators. (D) PLS-DA analysis in positive of brain metabolites. (E) Volcano plot showing differential metabolites between the STA and PT groups. (F) Heatmap showing correlations between differential metabolites and phenotypic indicators.

Metabolites serve as key mediators bridging gut-brain communication within the gut-brain axis (Deng et al., 2024). To further elucidate the mechanism of STA, untargeted metabolomic analyses were performed on fecal and brain tissue samples. Partial least squares discriminant analysis (PLS-DA) revealed clear separation between the PT and NC groups, as well as between the STA and PT groups in fecal samples under positive ion mode (Fig. 3A), indicating significant alterations in fecal metabolites. Differential metabolites between the STA and PT groups were further visualized using a volcano plot (Fig. 3B), where red and blue dots represent significantly upregulated and downregulated metabolites (*P* < 0.05), respectively. Endogenous metabolites with *P* < 0.05 were selected for heatmap visualization (Fig. 3C). Compared with the PT group, N-acetyldopamine and 5-Guanidinocaproic acid were significantly increased after STA supplementationt, whereas Adenine and Xanthine were significantly decreased.

As STA ultimately exerts its effects on the brain to alleviate stroke-related symptoms, brain tissue samples were further analyzed to explore the potential influence of gut microbiota on brain metabolism. The positive ion mode PLS-DA score plot (Fig. 3D) revealed pronounced metabolic alterations in brain tissues than in fecal samples. Differential metabolites between the STA and PT groups were identified using a volcano plot (Fig. 3E), and a heatmap constructed using the same criteria (Fig. 3F) showed that Palmitoylcarnitine and Adenine were significantly decreased, whereas Creatinine, Xanthine, and Valine were significantly increased following STA supplementation. These findings indicate that STA markedly modulates brain metabolic profiles, suggesting a potential metabolic basis for its neuroprotective effects.

**Fig. 4:**
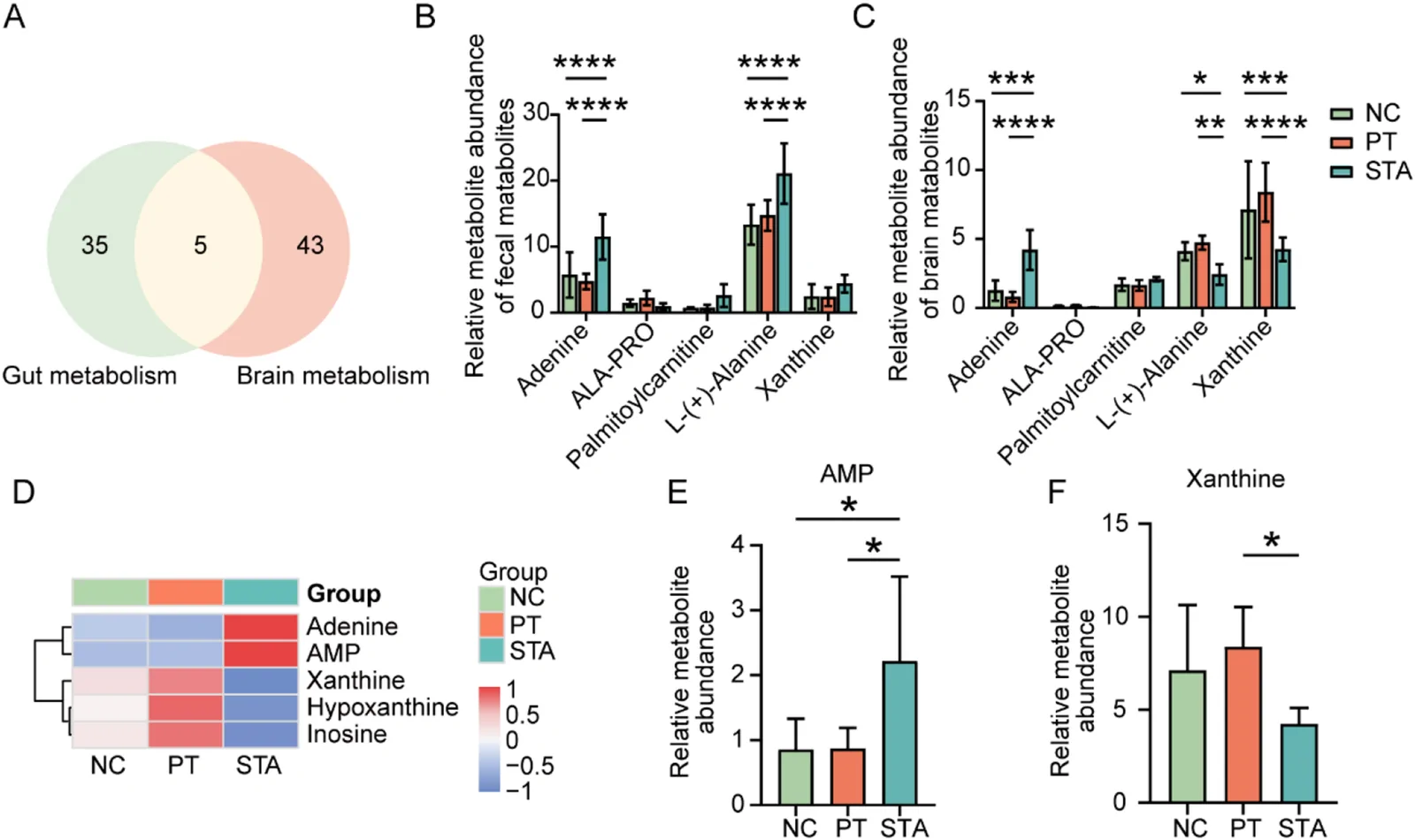
**Gut-brain common metabolites and purine pathway analysis.** (n = 6) (A) Venn diagram showing five shared differential metabolites between gut and brain tissues. (B) Expression levels of the overlapping metabolites in fecal. (C) Expression levels of the overlapping metabolites in brain (D) Heatmap of adenine-related purine metabolites in brain tissue. (E) Relative abundance of AMP in brain tissue. (F) Relative abundance of Xanthine in brain tissue. *P < 0.05, **P < 0.01, ***P < 0.001.****P < 0.0001.

To identify metabolites that may function as bridging signals within the gut-brain axis, we applied a Venn diagram analysis to the differential metabolites detected in fecal samples and brain tissues (Fig. 4A). The results revealed five overlapping metabolites shared between the two datasets. We further summarized the expression profiles of these five common metabolites in feces and brain tissue (Fig. 4B and C). Subsequent analysis showed that only adenine and ALA-PRO exhibited consistent variation trends in both feces and brain tissue. Published studies on ALA-PRO are limited, and fluctuations in small peptides containing proline are generally linked to the functional alterations of gut microbiota (Wu et al., 2022). Accordingly, we focused our follow-up investigation on adenine. A clustering heatmap was plotted to visualize the expression patterns of adenine-related metabolites in brain tissue, which are involved in purine salvage synthesis and catabolism (Fig. 4D). Quantitative verification demonstrated that AMP was markedly upregulated (Fig. 4E), while xanthine was significantly downregulated (Fig. 4F). Collectively, these findings indicate that peripheral adenine can penetrate into brain parenchyma and restore the homeostasis of nucleotide metabolism by facilitating the purine salvage pathway.

### 3.4 Transcriptomic profiling reveals stachyose regulates cerebral purine metabolism and neuroactive ligand-receptor signaling pathway in stroke mice

**Fig. 5:**
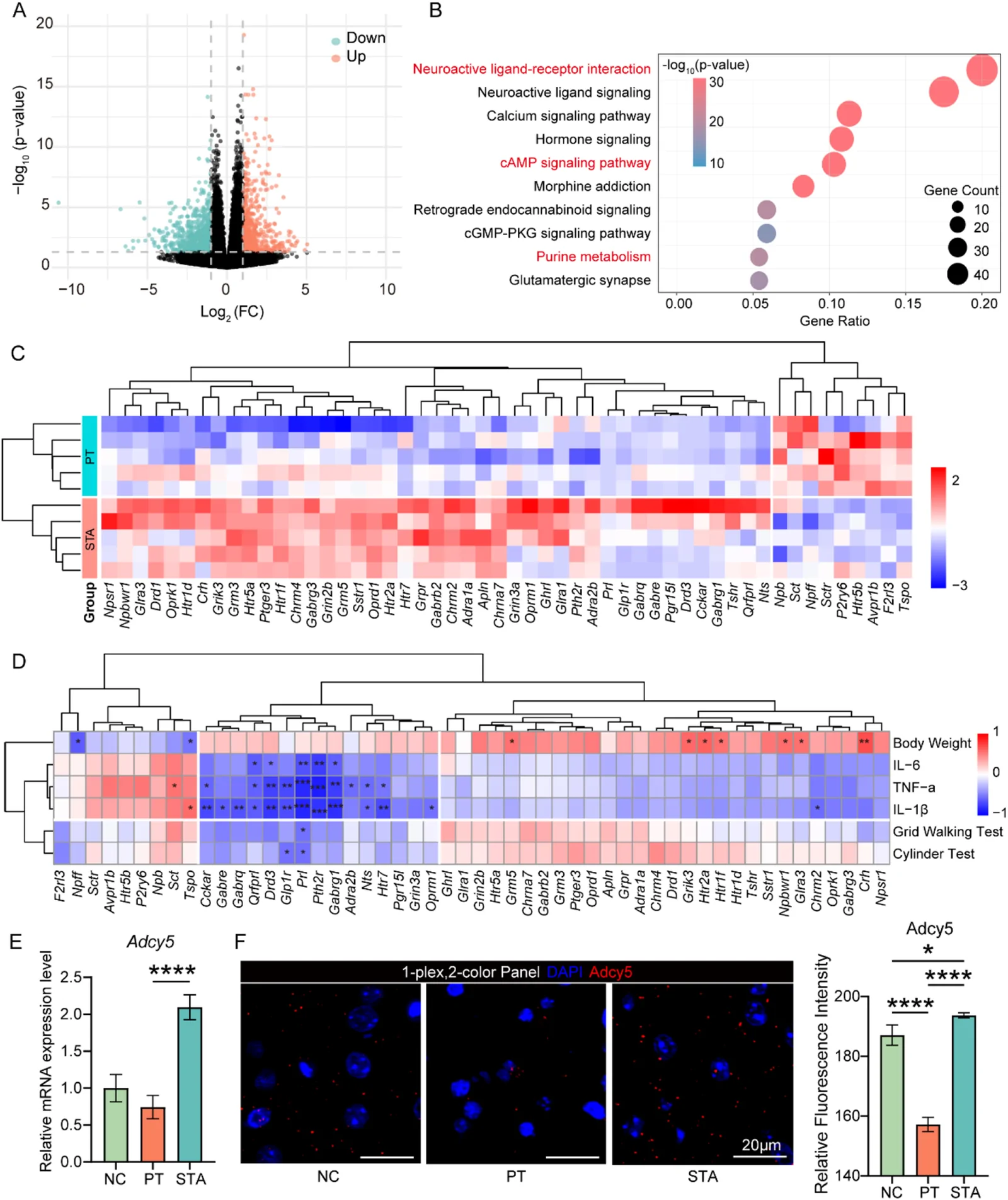
Brain transcriptome enrichment and verification of the purine metabolic cascade induced by stachyose intervention. (n = 5). (A) Volcano plot of DEGs between STA and PT group. (B) KEGG Pathway Enrichment Analysis of DEGs. (C) Heatmap showing the expression profiles of DEGs involved in the neuroactive ligand-receptor interaction pathway. (D) Spearman correlation analysis between representative DEGs and phenotypic indicator. (E) The mRNA level of *Adcy5*. (F) The immunofluorescence staining for Adcy5 protein. *P < 0.05, **P < 0.01, ***P < 0.001.****P < 0.0001.

To investigate the protective effects of STA on photothrombosis-induced stroke in mice, we used DESeq2 to compare the PT and STA groups and constructed a volcano plot to identify differentially expressed genes (DEGs) regulated by STA (Fig. 5A). The analysis revealed that, compared with the PT group, a total of 1283 DEGs were identified in the STA group, including 565 upregulated and 718 downregulated genes. To further elucidate the potential relationship between STA-associated transcriptomic alterations and the pathogenesis of stroke, Kyoto Encyclopedia of Genes and Genomes (KEGG) pathway enrichment analysis of the DEGs was performed and visualized using a bubble plot (Fig. 5B). The results showed that the DEGs were significantly enriched in the neuroactive ligand-receptor interaction pathway. DEGs involved in this pathway were subsequently visualized using a heatmap (Fig. 5C). Compared with the PT group, many genes were downregulated in the STA group, including *Tspo*, *Sct*, and *Npff*, whereas several genes were upregulated, such as *Htr1f*, *Drd3*, *Npbwr1*, *Glra3*, and *Prl*. To explore the roles of these genes in the protective effects of STA on stroke, we further performed Spearman correlation analysis between DEGs and phenotypic indicators (Fig. 5D). The results showed that the downregulated genes were negatively correlated with the body weight change rate and negatively correlated with inflammatory cytokine levels, suggesting that their downregulation contributed to body weight recovery and reduced inflammatory cytokine levels. In contrast, the upregulated genes were mostly positively correlated with body weight, and negatively correlated with inflammatory cytokine levels, contralateral hindlimb foot-fault rate, and forelimb support time asymmetry, indicating that their upregulation promoted functional recovery after stroke.

In addition, metabolomics identified adenine as a key differential metabolite, and KEGG enrichment of transcriptomic data highlighted the purine metabolism pathway. Notably, Adcy5 and Adcy9, which encode ATP-to-cAMP adenylyl cyclases, were significantly upregulated among the differentially expressed genes associated with the purine metabolism pathway. These findings imply that stachyose increases Adcy5-mediated adenylyl cyclase activity to accelerate cAMP production, enhancing cerebral cAMP signaling, a pathway also enriched in our KEGG analysis (Fig. 5B). RT-qPCR confirmed elevated *Adcy5* mRNA (Fig. 5E), and immunofluorescence verified its increased protein expression (Fig. 5F). Collectively, upregulated Adcy5 likely promotes cAMP synthesis and intracellular accumulation in brain cells. As a second messenger, cAMP may amplify the effects of multiple upregulated DEGs in the neuroactive ligand-receptor interaction pathway via downstream cascades. Through the bidirectional gut-brain axis, this mechanism may partially restore neurotransmitter transmission, synaptic signaling, and neural microenvironment homeostasis in the central nervous system.

### 3.5 Multi-omics integration analysis reveals the mechanism by which STA supplementation alleviates photothrombosis-induced stroke in mice

**Fig. 6:**
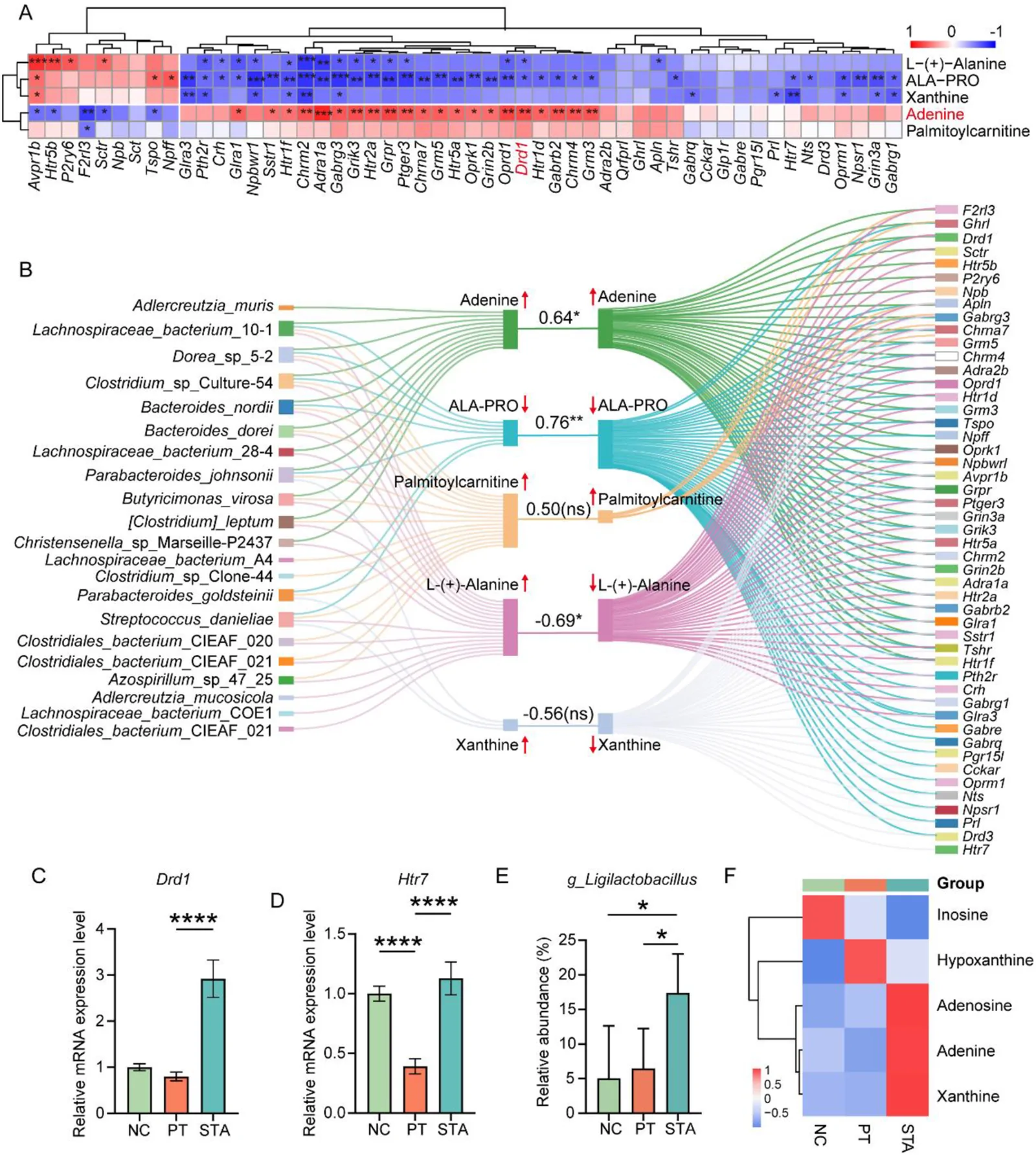
Correlation analysis across multi-omics datasets. (n = 5) (A) Spearman correlation analysis between DEGs associated with the neuroactive ligand-receptor interaction pathway and shared metabolites. (B) Sankey diagram illustrating the interactions among gut microbiota, shared metabolites, and DEGs. The mRNA level of *Drd1* (C) and *Htr7* (D). (E) Relative abundance of *g_Ligilactobacillus* across three groups. (F) Heatmap of adenine-related purine metabolites in feces. *P < 0.05, **P < 0.01, ***P < 0.001, ****P < 0.0001.

Integrative multi-omics analysis was further conducted to elucidate the protective mechanisms of STA against ischemic stroke. Spearman correlation analysis between brain tissue metabolites and differentially expressed genes enriched in the neuroactive ligand-receptor interaction pathway (Fig. 6A) demonstrated that the core metabolite adenine exhibited strong significant correlations with multiple genes within this pathway, including key receptor-coding genes such as *Drd1* and *Adra1a*. We subsequently explored the correlations among gut microbiota, pivotal metabolites, and differentially expressed genes involved in the neuroactive ligand-receptor interaction pathway (Fig. 6B). The correlation results revealed that gut strains participating in purine metabolism remodeling, such as *s_Bacteroides* dorei and *s_Lachnospiraceae* bacterium 10-1, were markedly associated with adenine. These observations indicated that specific gut microbial taxa are capable of modulating the biosynthesis and homeostasis of adenine, which further regulates the expression of genes in the neuroactive ligand-receptor interaction pathway and ultimately mediates the neuroprotective effect of STA on ischemic stroke. To validate the downstream targets, we quantified the transcriptional levels of G protein-coupled receptor coding genes *Drd1* and *Htr7* (Fig. 6C and D). The results showed that STA supplementation significantly upregulated the expression of *Drd1* and *Htr7*. These two genes likely participate in cAMP-dependent neurotransmitter signal transduction, thereby facilitating the restoration of neural plasticity and the reconstruction of neurological function in injured brain tissue.

In addition, we found that bacteria belonging to the genus *Ligilactobacillus* secrete adenine during their proliferation, and the relative abundance of this genus was remarkably elevated in the intestinal tract of STA-treated mice (Fig. 6E). Given that intestinal adenosine levels did not decrease noticeably after STA intervention, we inferred that the overall adenine metabolic pool was elevated collectively by the synergistic effects of multiple gut commensal microbes.

**Fig. 7:**
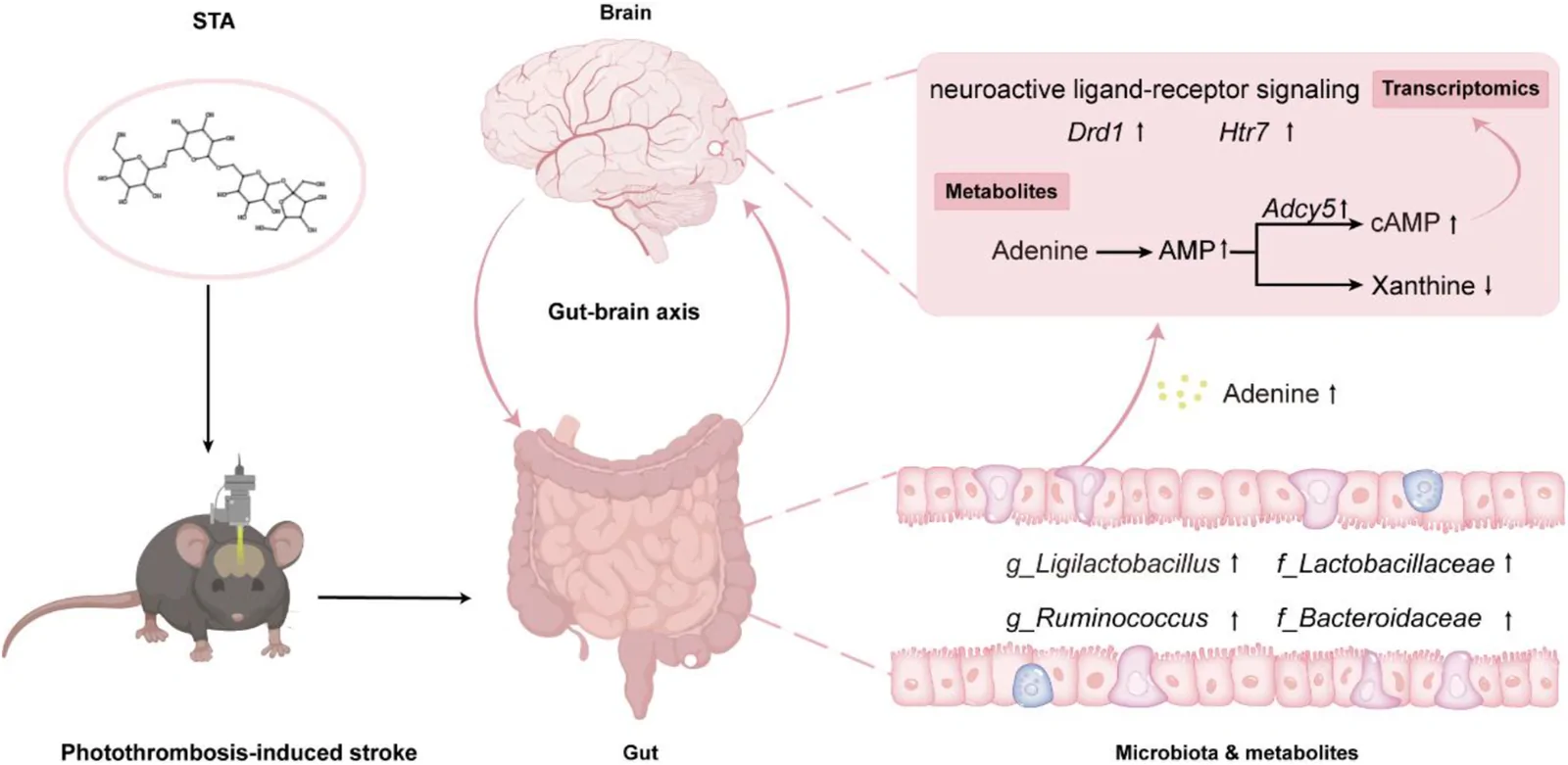
Proposed mechanism of the protective effects of STA on stroke.

In summary, these findings establish a potential mechanistic model underlying STA-mediated protection against ischemic stroke (Fig. 7). STA supplementation remodels the composition of gut microbiota, leading to a comprehensive shift in intestinal metabolic profiles. These gut-derived metabolites subsequently modulate brain metabolic homeostasis through gut-brain axis communication. The altered brain metabolites further govern the transcription of genes implicated in the neuroactive ligand-receptor interaction signaling pathway, ultimately exerting the therapeutic and neuroprotective effects of STA in ischemic stroke. This integrative multi-omics analysis uncovers a microbiota-metabolite-brain regulatory axis, which serves as the core molecular foundation for gut-brain crosstalk during stroke recovery.

## 4 Discussion

Stroke is one of the leading causes of death and long-term neurological disability worldwide (Feigin et al., 2014). Current therapeutic strategies are limited by narrow treatment windows and restricted patient eligibility, and many patients still experience high rates of disability (Saini et al., 2021). For ischemic stroke, current clinical management primarily relies on timely reperfusion therapies, while experimental studies have also explored pharmacological neuroprotective agents, such as edaravone (Yamashita and Abe, 2024) and minocycline (Lu et al., 2021; Wang et al., 2024); however, effective and widely applicable neuroprotective strategies remain limited. Therefore, exploring safe and effective novel intervention strategies is of great importance for the prevention and treatment of stroke. STA, a prebiotic oligosaccharide, has been shown to promote the growth of beneficial bacteria such as *Bifidobacterium* and *Lactobacillu*s, thereby improving the intestinal microbial environment (Kim et al., 2025). It may exert therapeutic effects on stroke by modulating gut microbiota through the gut-brain axis (Tan et al., 2021; Zheng et al., 2022). However, the role of STA in stroke and its underlying mechanisms remain largely unexplored. In this study, a multi-omics analysis strategy was employed to systematically evaluate the protective effects of STA on ischemic stroke and to investigate its potential mechanisms from the perspective of the gut-brain axis.

Our integrative multi-omics analysis identified adenine as a shared differential metabolite altered consistently in both intestinal contents and brain tissue (Fig. 4B and C). As a metabolite detected across the gut and brain compartments, adenine represents a plausible mediator of gut-brain communication, potentially transported via systemic circulation (Chen et al., 2025). Nonetheless, emerging evidence indicates that gut signals can also modulate brain function through direct gut-brain neural pathways (Aburto and Cryan, 2024; Cao et al., 2024). This alternative route was not evaluated in the current work and merits further exploration (Jameson et al., 2025). Additionally, adenine abundance was characterized using untargeted metabolomics. Although untargeted profiling enables broad metabolite screening, it lacks the quantitative precision of targeted approaches (Amer et al., 2023; Beger et al., 2024). Subsequent targeted metabolomics assays are therefore required to validate adenine fluctuations and clarify its systemic bioavailability and functional relevance to gut-brain signaling.

We further explored whether STA-driven gut microbial remodeling accounts for shifts in the intestinal adenine pool. STA supplementation reshaped the overall gut microbial community and enriched multiple Firmicutes-associated taxa, including *g_Ligilactobacillus* (Rodríguez et al., 2023), *g_Ruminococcus*(Liu et al., 2023; Yang et al., 2024), and *g_Bacteroides* (Guo et al., 2016) (Fig. 2I). Notably, *g_Ligilactobacillus* was markedly expanded after STA treatment, in line with prior reports describing its capacity to synthesize adenine during bacterial growth (Kasahara et al., 2023; Rimaux et al., 2011). However, the absence of a significant reduction in intestinal adenosine implies that elevated adenine cannot be explained by a single microbial species alone. Supporting this view, multi-omics correlation analysis revealed that several strains participating in purine metabolic reprogramming, such as *s_Bacteroides dorei* and *s_Lachnospiraceae* bacterium 10-1, were significantly correlated with adenine levels. These observations suggest that STA increases the intestinal adenine pool via coordinated changes in microbial community composition and metabolic function, rather than selective expansion of one adenine-producing taxon.

We then investigated the potential downstream molecular changes in the brain. Transcriptomic analysis showed that STA markedly regulated the neuroactive ligand–receptor interaction pathway. Among the altered genes, *Drd1* (Kern et al., 2015) and *Htr7* (Prasad et al., 2019) were further validated. DRD1 is a dopamine D1 receptor that is coupled to Gs proteins and can promote adenylate cyclase activity and cAMP signaling, pathways involved in neuronal survival, synaptic plasticity, and functional recovery after cerebral ischemia. Consistent with the involvement of cAMP-related signaling, Adcy5, which encodes adenylate cyclase 5, was also upregulated following STA supplementation. This finding suggests that enhanced ADCY5-associated cAMP signaling may contribute to the neuroprotective effects of STA, although direct changes in cAMP levels remain to be experimentally validated. HTR7 is a serotonin receptor that is also positively coupled to adenylate cyclase and has been implicated in the regulation of neuronal activity and neuroplasticity (Costa et al., 2018; Y. Huang et al., 2022). The increased expression of Drd1 and Htr7 following STA supplementation therefore suggests that modulation of neurotransmitter receptor signaling may contribute to the neuroprotective effects of STA after ischemic stroke. Importantly, the correlations observed between adenine and these receptor-related genes provide an association linking gut microbial metabolic remodeling with brain neurotransmitter signaling, but do not establish a direct causal relationship. Further experiments are required to determine whether adenine or its downstream metabolites directly regulate DRD1/HTR7 signaling and whether this contributes to the protective effects of STA.

Taken together, these findings support a potential gut-microbiota-metabolite-brain signaling framework in which STA remodels the gut microbial community, alters adenine metabolism, and is associated with changes in brain neurotransmitter receptor signaling. Although the present data do not establish a direct causal pathway, the identification of adenine as a gut-brain shared metabolite and its association with Drd1 and Htr7 provide potential mechanistic links between microbial metabolic remodeling and neural signaling. Future targeted metabolomic and functional studies will be necessary to clarify the contribution of adenine and define the precise route and molecular mechanisms underlying the gut–brain effects of STA.

Despite these findings, several limitations should be acknowledged. First, the present study was primarily conducted using animal models, and the findings require further validation in clinical populations. Second, although STA supplementation showed protective effects when administered before stroke induction and continued during the post-stroke period, the therapeutic efficacy of STA when initiated after stroke onset requires further investigation in dedicated post-stroke intervention studies. Third, It should be noted that the reagent-grade stachyose used herein does not fully recapitulate the natural food matrix, wherein co-existing dietary fibers and proteins may influence its digestive behavior and bioavailability. As such, this study provides mechanistic proof-of-concept data. Further validation using food-grade extracts or stachyose-rich soy foods is required to confirm the practical significance of these effects in real-world dietary scenarios. Forth, we agree that the relatively small sample size represents a limitation, particularly for integrative multi-omics correlation analyses, and future studies with larger cohorts are needed to validate these findings. In addition, a formal a priori power calculation was not performed before the experiments, which should be considered as a limitation of the current study.

Future studies should conduct multicenter randomized controlled clinical trials to further evaluate the clinical efficacy of STA in stroke patients. Moreover, functional experiments are required to clarify the regulatory mechanisms linking gut microbiota, microbial metabolites, and neural signaling pathways. In particular, future studies using antibiotic depletion, fecal microbiota transplantation (FMT), or germ-free animal models will be necessary to further establish the causal contribution of gut microbiota to the STA-mediated gut-brain regulatory axis. Expanding research in this area may provide a more solid theoretical foundation for the clinical translation of STA in stroke prevention and treatment.

## Author contributions

Zichen Zhang: Conceptualization, Animal experiments, Formal analysis, Bioinformatics analysis, Writing - Original draft preparation. Haoyan Zhang: Conceptualization, Animal experiments, Formal analysis, Bioinformatics analysis, Writing - Original draft preparation. Xueying Liu: Animal experiments, Formal analysis, Bioinformatics analysis. Shengxiang Wang: Animal experiments, Formal analysis, Graph plotting and visualization. Yichen Liu: Animal experiments, Formal analysis, Graph plotting and visualization. Peng Liu: Animal experiments, Formal analysis. Shangyong Li: Conceptualization, Writing - Original draft preparation. Jieting Zhang: Conceptualization, Funding acquisition, Writing - Original draft preparation, Writing - review and editing. Ningning He: Conceptualization, Graph plotting and visualization, Writing - Original draft preparation. Shihai Liu: Conceptualization, Writing - Original draft preparation.

## Ethics declaration

This study was conducted in accordance with the ARRIVE (Animal Research: Reporting of In Vivo Experiments) guidelines. This study was approved by the Ethics Committee of the Medical College of Qingdao University. (Approval No. QDU-AEC-2024780)

## Data availability

The authors confirm that the data supporting the findings of this study are available at PRJNA1446739 (NCBI). The data supporting this article are found within the text and the supplementary information file. Any additional data and the data that support the plots within this paper are available from the corresponding author upon reasonable request.

## Funding

This study was supported by the 10.13039/501100007129Natural Science Foundation of Shandong Province (No. ZR2022QH119).

## Declaration of competing interest

The authors have declared that no competing interest exists.
