# Supplementary material for: Stachyose supplementation attenuates ischemic stroke injury via gut-brain axis mediated adenine metabolism and neuroactive ligand-receptor signaling
Source: Curr Res Food Sci. 2026 Sep 10;13:101556. doi: 10.1016/j.crfs.2026.101556 (PMC13587060; doi:10.1016/j.crfs.2026.101556)
Supplement: Supplementary file 1 [file mmc1.docx]

**Supplementary Materials**

**Table S1. Overall PERMANOVA analysis of gut microbial community composition based on Bray-Curtis distances.**

| **Term** | **Df** | **SumOfSqs** | **R2** | **F_value** | **P_value** |
| --- | --- | --- | --- | --- | --- |
| Model | 2 | 0.9787 | 0.3343 | 3.0125 | 0.0020 |
| Residual | 12 | 1.9493 | 0.6657 | NA | NA |
| Total | 14 | 2.9280 | 1 | NA | NA |

*NA: not available

**Table S2. Pairwise PERMANOVA comparisons of gut microbial community composition among experimental groups.**

| **Comparison** | **Df** | **SumOfSqs** | **R2** | **F_value** | **P_value** |
| --- | --- | --- | --- | --- | --- |
| NC vs PT | 1 | 0.1322 | 0.0872 | 0.7644 | 0.7640 |
| NC vs STA | 1 | 0.6371 | 0.3399 | 4.1190 | 0.0140 |
| PT vs STA | 1 | 0.6989 | 0.3535 | 4.3739 | 0.0100 |

**Table S3. PERMDISP analysis of gut microbial community dispersion among experimental groups.**

| **Df** | **Sum Sq** | **Mean Sq** | **F value** | **Pr(>F)** |
| --- | --- | --- | --- | --- |
| 2 | 0.0037 | 0.0018 | 0.2721 | 0.7664 |
| 12 | 0.0809 | 0.0067 | NA | NA |

*NA: not available

**Table S4 . The primers sequence for RT-qPCR.**

| **Gene** | **Forward (5’-3’)** | **Reverse (5’-3’)** |
| --- | --- | --- |
| *GAPDH* | TGTAGACCATGTAGTTGAGGTCA | AGGTCGGTGTGAACGGATTTG |
| *Adcy5* | CTTGGGGAGAAGCCGATTCC | ACCGCTTAGTGGAGGGTCT |
| *Drd1* | GGGTGGTGAGTGATTGG | CTGTGAGGATGCGAAAG |
| *Htr7* | CACCTCCGCTCTTTCCTTCTG | CGTAGTTGATCTGTTCCCCAC |
| *TNF-α* | TATGGCCCAGACCCTCACA | GGAGTAGACAAGGTACAACCCATC |
| *IL-6* | TAGTCCTTCCTACCCCAATTTCC | TTGGTCCTTAGCCACTCCTTC |
| *IL-1β* | GAGCACCTTCTTTTCCTTCATCTT | TCACACACCAGCAGGTTATCATC |
